# Coating of Carbon Fiber with Polyhedral Oligomeric Silsesquioxane (POSS) to Enhance Mechanical Properties and Durability of Carbon/Vinyl Ester Composites

**DOI:** 10.3390/ma4091619

**Published:** 2011-09-21

**Authors:** Hassan Mahfuz, Felicia Powell, Richard Granata, Mahesh Hosur, Mujib Khan

**Affiliations:** 1 Ocean and Mechanical Engineering Department, Florida Atlantic University, Boca Raton, FL 33431, USA; E-Mails: fpowell1@fau.edu (F.P.); granatar@fau.edu (R.G.); 2 Materials Science and Engineering Department, Tuskegee University, Tuskegee, AL 36088, USA; E-Mail: hosurm@tuskegee.edu; 3 Department of Mechanical Engineering, University of Texas at El Paso, TX 79968, USA; E-Mail: mkhan@utep.edu

**Keywords:** nanocomposite, POSS, carbon fiber, vinyl ester, F/M interface

## Abstract

Our continuing quest to improve the performance of polymer composites under moist and saltwater environments has gained momentum in recent years with the reinforcement of inorganic nanoparticles into the polymer. The key to mitigate degradation of composites under such environments is to maintain the integrity of the fiber/matrix (F/M) interface. In this study, the F/M interface of carbon/vinyl ester composites has been modified by coating the carbon fiber with polyhedral oligomeric silsesquioxane (POSS). POSS is a nanostructured inorganic-organic hybrid particle with a cubic structure having silicon atoms at the core and linked to oxygen atoms. The advantage of using POSS is that the silicon atoms can be linked to a substituent that can be almost any chemical group known in organic chemistry. Cubic silica cores are ‘hard particles’ and are about 0.53 nm in diameter. The peripheral organic unit is a sphere of about 1–3 nm in diameter. Further, cubic structure of POSS remains intact during the polymerization process and therefore with appropriate functional groups, if installed on the fiber surface, would provide a stable and strong F/M interface. Two POSS systems with two different functional groups; namely, octaisobutyl and trisilanolphenyl have been investigated. A set of chemical and mechanical procedures has been developed to coat carbon fibers with POSS, and to fabricate layered composites with vinyl ester resin. Interlaminar shear and low velocity impact tests have indicated around 17–38% improvement in mechanical properties with respect to control samples made without the POSS coating. Saltwater and hygrothermal tests at various environmental conditions have revealed that coating with POSS reduces water absorption by 20–30% and retains the composite properties.

## 1. Introduction

Although carbon fiber reinforced polymer composites are widely used in various industries, their usage in marine applications is somewhat limited [1,2,3]. To date, the Navy has used traditional materials to construct their fleets; however, maintenance costs, fuel consumption, and warfare vulnerabilities has renewed the development of composite fabrication [4]. The durability of composites in moist environments has been a limiting factor in capitalizing on the structural advantages of composite vessels. Since epoxies are not as stable as vinyl ester in salt water environment, recent thrust has been to develop carbon/vinyl ester composites for marine structures. Several studies have been conducted investigating the effects of water on the mechanical properties of carbon/vinyl ester composites [5,6,7,8,9]. The mechanical and thermal analysis of all these studies indicated that the primary area of failure was the fiber/matrix (F/M) interface due to water degradation. As the F/M interface is primarily responsible for load transfer from the matrix to the fiber, its degradation signals initial failure of the composite. The objective of this investigation is to improve this F/M interface by coating the carbon fiber surface with polyhedral oligomeric silsesquioxane (POSS).

Modification of the fiber surface has been considered as a method of improving the fiber/matrix bonding [10,11,12]. Studies by Langston [13] and Verghese *et al.* [14] have shown that fiber surface modification improves the interface properties of carbon/vinyl ester composites. However, an improvement across the range of mechanical, thermal and hygrothermal behavior of composites was not observed.

To address this issue, we are investigating nanoscale inclusion at the carbon fiber surface. Nanoparticles, such as POSS, should be ideal for such coating of the fiber since the physical dimension of POSS is in nanometer range and is expected to enhance bonding at the interface. POSS is an inorganic-organic hybrid nano silica engineered to strengthen composites, as well as, promote hydrophobicity, which is ideal for marine applications. The advantage of using POSS is that silicon atoms can be linked to a substituent that can be almost any chemical group known in organic chemistry. POSS chemically bonded with thermoset polymers either enhances or reinforces the mechanical and thermal properties of the neat material system [15]. Recent studies by Zhang [16] have demonstrated 25–27% improvement of the interlaminar shear strength with various POSS products. It is therefore expected that if POSS particles, with appropriate functional groups, can be installed on the carbon fiber surface, they would provide a stable and strong F/M interface [17,18].

## 2. Experimentation

Two types of carbon/vinyl ester nanocomposites were manufactured; one with 1.0 wt % loading of octaisobutyl-POSS (Octa), and the other with 0.2 wt % of trisilanolphenyl-POSS (TriS). POSS particles were procured from Hybrid Plastics (55 W.L. Runnels Industrial Drive, Hattiesburg, MS 39401). As-received carbon fiber cloth (Vectorply, 3500 Lakewood Dr. Phenix City, AL, 36867) was cut into panels of 12 in × 12 in and placed in stacks of 6 plies into a Ziplock^®^ bag. Each Ziplock^®^ bag was filled with 240 mL of acetone, then the air bubbles were removed prior to sealing the Ziplock^®^ bag. The plies were soaked in acetone for 16 hours in order to remove the sizing from the fiber surface. Acetone was evaporated in a cooling oven preheated to 100 °C.

A liquid media was produced to de-agglomerate POSS particles and provide a medium for attaching the particles to the carbon fiber. The manufacturer specified two solvents; hexane for Octa and ethanol for TriS. The POSS product and respective solvent were mixed by a VDI-24 homogenizer at 2400 rev/min for 5 minutes. The amount of solvent in each batch was of sufficient quantity such that the fiber plies could be fully soaked. Weight percent of POSS was calculated based on the weight of the carbon fiber to be coated. Since the homogenizer was not able to completely de-agglomerate the particles, the admixture was then subjected to ultrasound cavitation to achieve appropriate dispersion of POSS particles. The solution was submerged in a 5.5 °C cooling bath and was sonicated for an hour and 30 minutes at 45% amplitude using the VCX 500 Vibra Cell Liquid Processor.

To coat carbon fibers with POSS, fiber plies were individually inserted in a Ziploc^®^ bag with 40 mL of the solution for each 6 in × 6 in ply. In case of each 12 in × 12 in ply, 140 mL of the solution was used. To ensure complete soaking, air bubbles were removed prior to sealing the bags. Each Ziploc^®^ bag was then secured to a standard massage pad set on high vibration mode for a duration of three hours. Residual solvent was then evaporated by keeping it in an cooling oven (preheated to 100 °C) for 30 minutes. Coated carbon fibers were next used to fabricate composite panels.

Traditional hand lay–up method followed by compression molding was used to fabricate the various categories of panels. Three categories of composite panels were fabricated using Derakane 8084 vinyl ester resin (J.I. Plastics 19233 Plank Road Zachery. LA 70791). The three types of panels were: (i) - fiber coated with 1.0 wt % octaisobutly; (ii) - fiber coated with 0.2 wt % Trisilanolphenyl; and (iii) - control panels with acetone treated carbon fiber. The resin was formulated as specified by the manufacture, namely, Ashland Inc. The process began with pouring of resin on the mold face. A single ply was stacked on top followed by the addition of resin spread over the entire ply. This sequence was repeated until all the required plies had been applied. The top mold plate was stacked at the end completing the hand lay-up operation. The mold was then clamped and allowed to cure. After curing for 24 hours the mold was debulked, and the panel was machined to extract samples for mechanical and hygrothermal analyses.

Short beam shear tests were carried out on a Z050 Zwick/Roell machine at a crosshead speed of 1.3 mm/min at ambient temperature. ASTM D2344 standard was followed to conduct the short beam shear tests. Span-to-thickness ratio was maintained at 4. Sample dimensions were 30 mm (length) × 10 mm (width) × 5 mm (thickness). Five specimens were tested in each category.

Low velocity impact tests were conducted using a Dynatup 8210 machine with a 12.5 mm diameter hemispherical tup. Impact energy was 30 J. The weight of the crosshead was 6.39 kg [19]. Specimen size was 2.8 mm × 101.6 mm × 101.6 mm. Each specimen was clamped into the sample holder at the bottom of the testing machine with a circular support spanning 75 mm. A GRC 930-I data acquisition system was used to compute the load, energy, velocity, and displacement during each impact. Three specimens were tested for each category.

For durability tests, composite samples were exposed to saltwater at room temperature, saltwater at 40 °C, and 85% humidity at 50 °C for six weeks. Thirty-six short beam shear specimens were used in this investigation. Specimens from each category of panels were weighed prior to immersion. Samples were weighed periodically to measure the rate of water absorption. Initially, the samples were weighed every 3–4 days followed by wider intervals up to a week. Water absorption was higher initially, and then leveled off as the samples became saturated. Percentage of weight change was determined as:

(1) $$M\%=\frac{M_{t}-M_{o}}{M_{0}}\times 100$$

where *M_t_* is the weight at time *t* and *M_0_* is the initial dry weight [7,20]. After environmental exposure, five specimens from each composite system were tested in short beam shear under two conditions; one is immediately after taking them from the testing environment (wet condition) and the other after drying them for several days in ambient conditions. Shear strength values were then compared with those obtained prior to environmental exposure.

## 3. Results and Discussion

### 3.1. Fiber Surface Analysis

**Figure 1:**
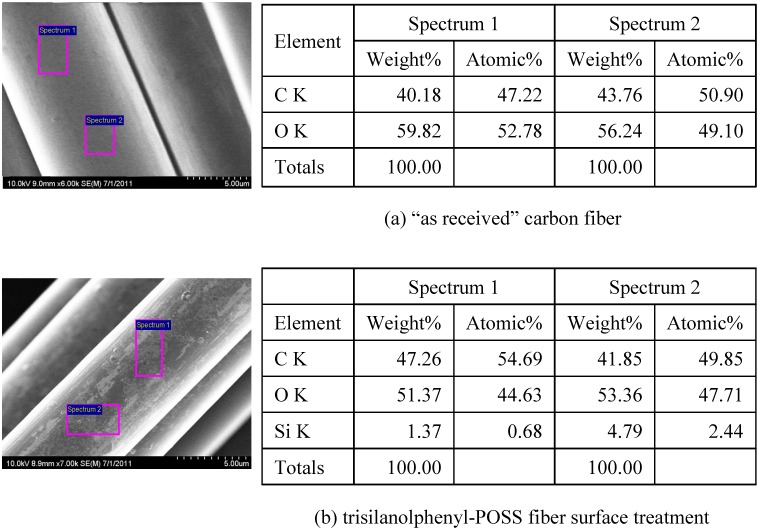
SEM images and EDS anaylsis of carbon fibers: (**a**) “as-received” and (**b**) POSS Treated.

In an attempt to assess the success of coating, SEM micrographs of fiber surfaces were examined as shown in Figure 1. Figure 1a shows the surface of as-received carbon fiber. The figure indicates a very clear surface. EDS studies at multiple sites have shown presence of elemental C and O. The oxygen may have come from oxidation of the fiber surface by the manufacturer to promote bonding. Weight percentage of these elements varied slightly at three locations examined on the fiber surface. No trace of silicon (Si) was observed. Fiber surface treated with POSS is shown in Figure 1b. Attachment of POSS coating on the fiber surface is quite evident. As seen in the SEM micrograph, the coating is more or less uniform on the surface. EDS studies have detected Si indicating the success of our coating methodology. Although we did not determine what chemical bonds were created between fiber and the POSS, the coating is smooth and adhering to the fiber. The reason for such adherence is coming from the silanol functional group that has been installed in the trisilanolphenyl-POSS. Silanol has Si atom at the core with OH functional groups at the outside which can interact with carbon fiber substrate and form Si–O–C chemical bonds. On the other hand, hydroxyl groups (OH) of silanol can also react with unsaturated C (reactive sites) present in vinyl ester molecules forming similar chemical bonds resulting in an intimate fiber/matrix interface.

### 3.2. Interlaminar Shear and Low Velocity Impact Tests

**Figure 2:**
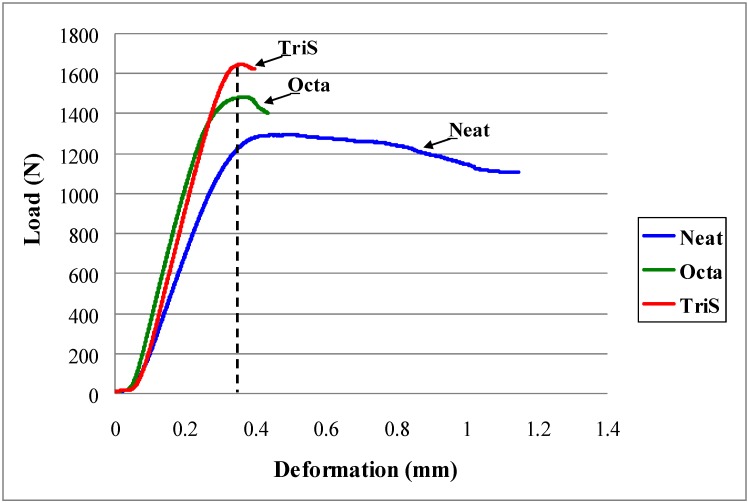
Short beam shear testing results.

Two types of mechanical tests were performed to see if there was any improvement in properties due to POSS coating of the fiber. Short beam shear and low velocity impact tests were conducted. Short beam shear tests were meant to measure the interlaminar shear strength of the composite. Since the span/depth ratio was low the tests allowed specimens to fail under in-plane shear. Short beam shear tests were done according to ASTM standard. Representative load-displacement curves for three categories of samples are shown in Figure 2. The curve for the neat specimen portrays an uncontrolled displacement after it reaches the maximum load. A distinct failure is not obvious until the specimen reaches the maximum displacement limit. On the other hand, clear failure of the composite is quite evident in the curves of Octa and TriS. The failure is almost brittle in nature. In both cases, the load reaches to a maximum and then failure occurs immediately after. Deflection to failure is much smaller compared to that of neat. The initial slope of both the curves is also much steeper than that of the neat. If tangent modulii were to be calculated, they would be much higher with the POSS-coated specimens. Calculated values of average strength with corresponding standard deviation are shown in Table 1. It is seen in Table 1 that interlaminar shear strength of the composite has increased by 17% and 25%, respectively with the coating of Octa and TriS. We attribute such increase in strength solely to POSS. Data in the table also suggests that TriS provides a better coating than Octa. Stiffening of the composite also corresponds to previous studies conducted with other nanoparticles corroborating the fact that any inorganic inclusion in the composite will tend to increase modulus and reduce fracture strain [21,22]. However if we look at the three curves up to the yield point, an important feature is revealed. Although it is not a standard procedure to measure modulus of resilience (toughness) from flexure tests, one can still have a good estimate of energy absorption up to yield from Figure 2. For example, a vertical line at 0.35 mm displacement up to the tip of each curve would clearly indicate that the area under the curve which, in essence is energy absorption, has increased significantly with POSS coated samples. Increase in strength and energy absorption thus demonstrates the effectiveness of our approach.

**Table 1:** Comparison of interlaminar shear strength.

Panel Type	Carbon Fiber wt %	Shear Strength (MPa)	Gain/Loss (%)
Neat	---	18.38 ± 0.72	---
Octa	1.0%	21.51 ± 0.73	17.03
TriS	0.2%	23.06 ± 1.82	25.48

**Figure 3:**
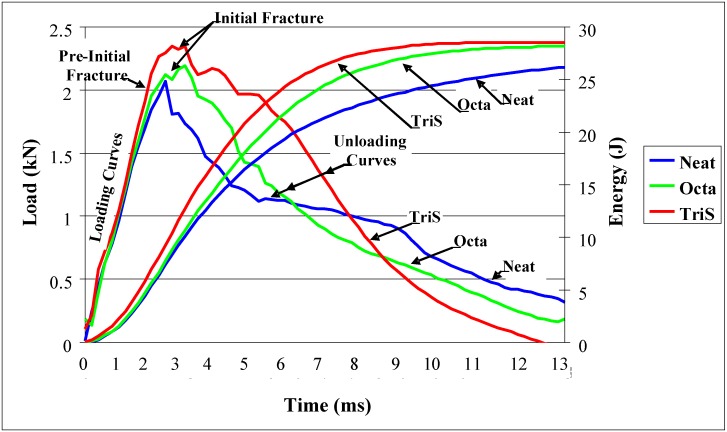
Low velocity impact testing results.

In the next step, samples were tested under low velocity impact. As the tup tends to penetrate during impact, energy is absorbed and dissipated from the point of impact. Absorption of energy will depend on delamination, matrix cracking, and interface separation. On the other hand, tensile wave speed through the composite will dictate the rate of dissipation of energy. Tensile wave speed through the composite in turn will depend on the modulii of the matrix and fiber, F/M matrix interface, and density of the composite. In other words performance under low velocity impact, in addition to energy absorption characteristics of the composite, will also reflect the integrity of the F/M interface modified by POSS. Load and energy *versus* time curves from the impact tests are shown in Figure 3. Load curves are to the left rising to a peak and then unloading to almost zero load level. Energy curves are seen to be rising steadily and leveling off at around 29 joules. Load and energy curves in Figure 3 are shown for three categories of samples; neat, Octa, and TriS. Load curve for the neat indicates a linear ramping up to the maximum load point without any load drops. Since load drops during the ascending stage of the load curve are indicative of the existence of incipient damage point (IDP). The IDP can be detected by a change of slope in the loading portion of the load *vs.* time curve [23]. The curve for the neat suggests that there is no IDP. However in case of TriS and Octa there are slight load drops just before reaching the peak meaning that IDP is present. Before IDP, the load drop indicates initial matrix damage in the early stage of the impact event, and after IDP the load drop indicates delamination and F/M interface failure. Since load drops are very small in these two cases, presence of IDP suggests that there is modest delamination and interface failure with the POSS modified samples. The maximum load point (MLP) provides the peak value that a panel can tolerate under a particular impact event before undergoing major damage. Usually at the MLP a major fiber breakage occurs through the thickness [24] and this breakage normally starts from the back face (tension side) followed by a penetration on the front face (compression side). Peak values for neat, Octa and TriS are 2.14, 2.26 and 2.51 KN, respectively. It is clear that at this energy level (29J) TriS specimens can withstand higher impact load as compared to neat and Octa (Table 2). The load curve after MLP is denoted as unloading curve. It is seen in Figure 3 that unloading curves for all three categories gradually descend with more or less smooth load drops until contact ceases. However, there are some differences between the three unloading curves. In case of neat, unloading from MLP is very sudden up to about 4 ms and then it gradually drops to 0.4 KN load. Such sudden drop indicates more fiber breakage at the tensile side of the coupon. In case of Octa and TriS the rate of unloading is relatively smoother without any sudden change in slope suggesting a gradual progression of failure. It is also noticed in Figure 3 that loading curves for the three systems are not symmetric about MLP, that is they have shorter time (~2.5 ms) for fracture initiation (*i.e.*, time required to reach the peak load) but longer propagation time. However with Octa and TriS samples, it is observed that there is also a initial fracture region before the load reaches the peak. This initial fracture region is followed by pre-initial fracture as indicated in Figure 3. This two step fracture process with POSS modified samples during the loading apparently sets the stage for gradual damage progression, especially in the case of TriS samples.

**Table 2:** Comparison of low velocity impact parameters.

Impact Parameters at Peak Load	Fiber Sizing Agent				
	Neat	Octa	G/L*	TriS	G/L*
Load (kN)	2.14	2.26	5.61	2.51	17.3
Energy (J)	8.22	8.45	2.80	11.4	38.3
Deflection (mm)	6.79	6.71	−1.18	7.85	15.6
Time to Peak (ms)	2.37	2.35	−0.84	2.81	18.6
Front Damage (m^2^)	6.02	4.70	21.80	4.27	29.03
Back Damage (m^2^)	4.32	2.49	42.40	2.05	52.63

The energy *versus* time for all three curves are seen to be leveling off at constant values between 26 and 29 joules. The point where the load returns to zero or the energy curve levels off to a constant value is denoted as total point (TP). The energy corresponding to TP represents the total energy absorbed by the panel and indicates the end of the impact events. The total energy absorbed at 29 joules impact level for three categories are shown in Table 2. Energy values in Table 2 were calculated from the energy curves corresponding to peak loads as shown in Figure 3. These peak loads occurred around 2.5 ms and accordingly energy absorptions were 8.22 J and 8.45 J, respectively for neat and Octa as seen in Table 2. On the other hand, energy absorbed by TriS is 11.4 J which is 38% higher than that of the neat. This is a clear demonstration that carbon fiber coated with trisilanolphenyl-POSS will have superior impact performance compared to the other two systems.

### 3.3. Moisture Analysis

**Figure 4:**
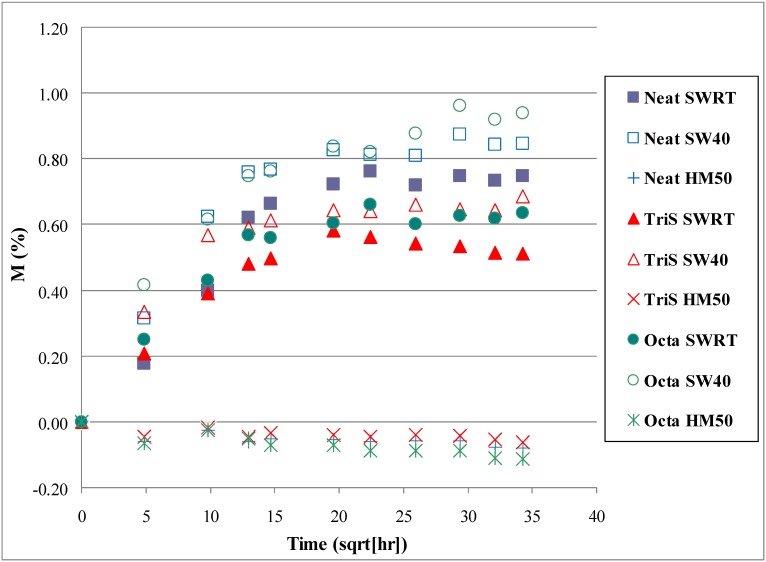
Percentage of weight change in saltwater 40 °C.

Having demonstrated that POSS improves mechanical properties in dry conditions, composite samples similar to those of short beam shear coupons were exposed to moist environments. For six weeks, composite samples were immersed in three different environments: saltwater at room temperature (SWRT), saltwater at 40 °C (SW40), and in 85% humidity at 50 °C (HM50). In each environment, a total of 36 specimens were exposed comprising of 12 samples from each type of panel: Neat, Octa, and TriS. The moisture absorption data for all categories of samples are shown in Figure 4 and Table 3. It is observed in Figure 4 that moisture uptake for samples at SWRT and SW40 increases gradually with time and somewhat levels off towards the end of the six week period. It is also noticed that percentage gain in weight (M%) also increases as one switches from SWRT to SW40 condition. This suggests that as water temperature increases so does the amount of water absorption in the composite. But overall it is noticed that the moisture gain is very small—less than 1% of the original weight of the respective samples. In the case of humidity exposure, *i.e.*, under HM50, it is observed that none of the specimens absorb any moisture as data points hover around the 0.00% line (Figure 4). A comparison among the three systems is shown in Table 3. The table lists percentage gain for each category of samples under SWRT and SW40 conditions. Since there is no weight change under HM50, it is omitted in Table 3. Last two columns of Table 3 compare weight change of Octa and TriS with that of neat. It is observed under SWRT that Octa gains 0.64% weight as compared to that of neat (0.75%). This means that Octa absorbs 15% less water when compared with neat. This value is depicted in Gain/Loss column for Octa in Table 3. On the other hand, weight change for TriS is 0.51% meaning that it absorbs 32% less water with respect to neat specimens. As condition changed to SW40 *i.e.*, at higher temperature, Octa absorbed more water (0.94%) than the neat (0.85%). But that was not the case with TriS, it still absorbed less water (0.68%) than the neat. Amount of water absorption was 20% less compared to neat as indicated in the table. This again shows clearly that TriS coating is very effective in enhancing the durability of carbon/vinyl ester composites under salt water environment.

**Table 3:** Comparison of weight change in moist and saltwater environments.

Condition	Percentage of Weight Change (%)			Gain/Loss w.r.t Neat (%)	
	Neat	Octa	TriS	Octa	TriS
SWRT	0.75	0.64	0.51	15	32
SW40	0.85	0.94	0.68	−11	20
HM50	-	-	-		

**Figure 5:**
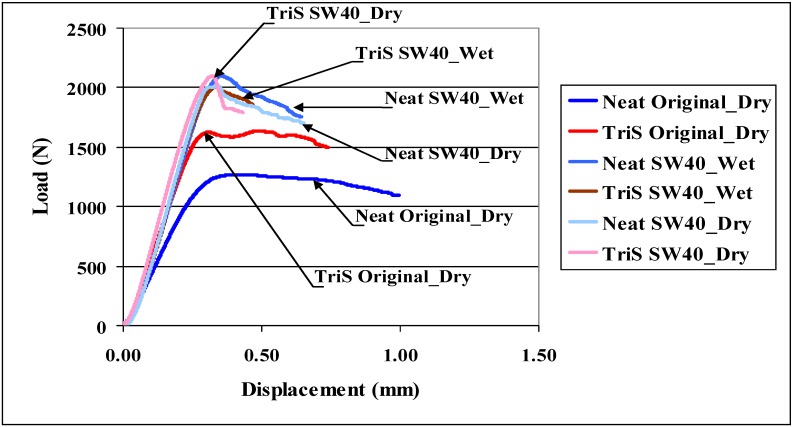
Seawater 40 °C short beam shear testing results.

In an attempt to assess the degradation of strength after environmental exposure, samples were tested in short beam shear at two stages; one category was tested immediately after the end of six week period while specimens were still wet (SW40_Wet), and the other was dried for two weeks at ambient temperature (SW40_Dry) and then tested. Although short beam shear tests were performed after all three environmental exposures, test data for SW40 are shown in this paper. Since the performance of TriS samples were most significant, short beam shear data for TriS only are shown in Figure 5. Two sets of curve–one for neat and the other for TriS are shown. Each set of neat and TriS has three curves for Original_Dry, SW40_Wet, and SW40_Dry. Strength data from these tests are shown in Table 4. It is evident from Table 4 that shear strength of the composite has increased rather than decreasing after SW40 exposure. Similar was the case with other conditions. This was true also with Octa. It is observed that in case of neat there is substantial gain in strength from 18 MPa to about 29 MPa after exposure. Gain in strength with TriS was however, moderate from 23 MPa to about 28–30 MPa. There is no significant change whether specimens are tested immediately after or after two weeks of drying. In any case, it was surprising to note that instead of loosing strength, composites were in fact gaining strength after the exposure. This trend was more prominent as the exposure temperatures increased from RT to 40 °C and then to 50 °C. Probable explanation for such behavior is that during environmental exposure, the resin was in fact still curing. Based on this observation, Original_Dry samples were post cured for at 90 °C for 2 hours as specified by the manufacturer. The resulting shear strength was 33 MPa, indicating that the composites were curing in the moist environments.

**Table 4:** Comparison of interlaminar shear strength after environmental exposure.

Shear Strength (MPa)			
Specimens	Original_Dry	SW40_Wet	SW40_Dry
Neat	18.38 ± 0.72	29.29 ± 1.35	29.58 ± 1.07
TriS	21.51 ± 0.73	28.64 ± 1.56	30.30 2.16

## 4. Summary

A chemical procedure has been developed to install POSS on the carbon fiber surface. The procedure is simple and involves a few low cost steps. SEM and EDS studies have demonstrated that the treatment of the fiber surface is indeed successful. Two types of POSS, namely, octaisobutyl and trisilanolphenyl have been investigated. Mechanical tests have indicated that both interlaminar shear strength and low velocity impact strength have improved by the reinforcement of POSS. However, the improvement was more significant with TriS in each case since TriS had compatible functional groups attached to its core silica molecule.

Environmental exposure experiments have revealed that water uptake during a six week period of time was minimal. Although water absorption was insignificant, it has been shown that composites with TriS performed the best under each of the exposure conditions. Mechanical tests, such as short beam shear test, of samples after exposure to environmental conditions have shown that there is no degradation in shear strength. In some cases, it has rather shown some improvement. This was true with neat, as well as with POSS reinforced composites. However, improvement with POSS was consistent as it was observed under dry conditions. It also suggests that while under environmental exposure, the resin was in fact curing rather than deteriorating within the six week time period, especially at elevated temperature. This explains the minimum amount of water absorption and slight increase in shear strength. Follow up experiments with post cured specimens have confirmed the assertion.
